# Exposure-Based Cognitive Behavior Therapy for Children with Abdominal Pain: A Pilot Trial

**DOI:** 10.1371/journal.pone.0164647

**Published:** 2016-10-13

**Authors:** Maria Lalouni, Ola Olén, Marianne Bonnert, Erik Hedman, Eva Serlachius, Brjánn Ljótsson

**Affiliations:** 1 Department of Medicine Solna, Clinical Epidemiology Unit, Karolinska Institutet, Stockholm, Sweden; 2 Stockholm Health Care Services, Stockholm County Council, Sweden; 3 Department of pediatric gastroenterology and nutrition, Sachs’ Children’s hospital, Stockholm, Sweden; 4 Department of Clinical Neuroscience, Division of Psychology, Karolinska Institutet, Stockholm, Sweden; 5 Department of Clinical Neuroscience, Centre for Psychiatry Research, Karolinska Institutet, Stockholm, Sweden; University of Nottingham, UNITED KINGDOM

## Abstract

**Background:**

Children with pain-related functional gastrointestinal disorders (P-FGIDs) have an increased risk for school absenteeism, depression, anxiety and low quality of life. Exposure-based cognitive behavior therapy (CBT) has shown large treatment effects in adults with irritable bowel syndrome, but has not been tested for children 8–12 years with P-FGIDs.

**Aim:**

The aim of this trial was to test the feasibility, acceptability and potential efficacy of a newly developed exposure-based CBT for children with P-FGIDs.

**Method:**

The children (n = 20) with a P-FGID, were referred by their treating physicians. The participants received 10 weekly sessions of exposure-based CBT and were assessed at pre-treatment, post-treatment and 6-month follow-up.

**Results:**

Children improved significantly on the primary outcome measure pain intensity at post (Cohen’s *d* = 0.40, p = 0.049) and at 6-month follow-up (Cohen’s *d* = 0.85, p = 0.004). Improvements were also seen in pain frequency, gastrointestinal symptoms, quality of life, depression, anxiety, school absenteeism and somatic symptoms. Improvements were maintained or further increased at 6-month follow-up. The children engaged in the exposures and were satisfied with the treatment.

**Conclusions:**

Exposure-based CBT for children with P-FGIDs is feasible, acceptable and potentially efficacious.

## Introduction

Pain-related functional gastrointestinal disorders (P-FGIDs) are characterized by abdominal pain without an organic explanation. Pain is sometimes accompanied by other gastrointestinal symptoms such as bloating, nausea and fecal disturbances. A worldwide prevalence of 13.5% was reported in a recent meta-analysis [1]. The most common P-FGIDs are irritable bowel syndrome (IBS), functional abdominal pain (FAP) and functional dyspepsia (FD), defined according to the Rome III criteria [2]. Patients with IBS have marked fecal disturbances accompanying the pain, whereas patients with FD have pain located to upper abdomen and patients with FAP experience pain in the lower or all of the abdomen [2].

The potential impact on children’s lives is considerable as P-FGIDs are associated with school-absenteeism, depression, anxiety and poor quality of life [3, 4]. For many children the symptoms sustain into adulthood [5]. Neither medication nor diet have been proven to be effective for children with P-FGIDs [6, 7], but different cognitive behavioral therapies (CBT) show promising results [7]. However, it is unclear which CBT components are effective [8].

In adults, gastrointestinal specific anxiety and avoidance of symptom-provoking stimuli have been shown to play a key role in the maintenance of IBS [9]. Children with P-FGIDs show the same behavioral pattern, with avoidance of situations in which they are afraid of having symptoms, and avoidance of symptom-provoking stimuli [10]. Exposure therapy is a mainstay in CBT for anxiety disorders [11] and using exposure exercises to reduce avoidance behaviors could be an effective intervention for children with P-FGIDs. In exposure therapy the child willingly provokes symptoms and approaches feared situations and aversive stimuli in order to decrease fear of symptoms and avoidant behaviors. Exposure-based CBT in adults with IBS has demonstrated efficacy with large effect sizes in several studies [12–15], and has also been tested for adolescents with P-FGIDs with promising results [16].

Parental attention to children’s pain complaints has been shown to increase the child’s perception of pain [17] and treatments that target parental behavior have shown effects on children’s pain symptoms [18–20]. Therefore, exposure-based CBT for children with P-FGIDs should include interventions that aid parents to decrease attention to pain complaints, reinforce healthy behaviors and facilitate exposure exercises. To the best of our knowledge, this is the first study of exposure-based CBT for young children with P-FGIDs and their parents.

The aim of the study was to investigate the feasibility, perceived usefulness and potential efficacy of a novel exposure-based CBT protocol for children 8–12 years with P-FGIDs and their parents.

## Methods

### Participants

The sample comprised 20 children 8–12 years (14 girls, 6 boys). Five of the children had separated parents and 15 lived with both parents. For baseline characteristics, see Table 1. Inclusion criteria were: (a) age 8–12 years, (b) living in Stockholm County, (c) a diagnosis of IBS, FAP or FD according to the Rome III criteria, (d) less than 40% school-absenteeism (e) stable dosage since at least three months if using psychopharmacological medications, (f) no other ongoing psychological treatment and (g) being able to speak and understand Swedish (child and at least one parent). Children with medical conditions that explained their abdominal symptoms (e.g. celiac disease) or severe psychosocial or psychiatric problems were excluded and referred to other treatments.

**Table 1:** Patient characteristics at baseline (N = 20).

Age, m (SD)	10.8	(1.2)	8–12 (range)
Duration abdominal problems m (SD)	3.7	(2.1)	0.5–8 (range)
Ethnicity, n			
Born in Sweden	20	100%	
At lest one parent born outside Sweden	5	25%	
ROME-III diagnosis, n			
Irritable bowel syndrome	13	65%	
Functional abdominal pain	7	35%	
Functional dyspepsia	0		
Referring care unit, n			
Primary care	2	10%	
Secondary care	12	60%	
Tertiary care	6	30%	
Heredity, n			
At least one parent with abdominal problems	11	51%	
Medication, abdominal symptoms ^a^, n	4	20%	
Psychiatric symptoms, n			
Depression CDI≥13 ^b^	2	10%	
Anxiety SCAS≥33 ^c^	5	25%	
School absence due to pain	17	85%	
Parents (N = 39)			
Education, n ^d^			
High School < 3 years	5	13%	
High School ≥ 3 years	9	23%	
University < 3 years	5	13%	
University ≥ 3 years	20	51%	

^a^ Omeprazol, Gaviscon, Macrogol & Novalucol.

^b^ Cut off indicating diagnostic level of depression [21].

^c^ Cut-off indicating diagnostic level of anxiety [22].

^d^ High school education < 3 years = vocational education, ≥ 3 years preparing for university studies. University ≥ 3 years represents a Bachelor’s degree or above.

### Procedure

The study used a within-group design, where all participants received exposure-based CBT for 10 weeks. Participants were referred to the study by physicians working in primary, secondary and tertiary care units in Stockholm County Council. The physicians had to conclude that symptoms were functional, with tests excluding inflammation and celiac disease, before referring to the study. The study was conducted at the Child and Adolescent Psychiatry Research Centre, a research unit within the child and adolescent psychiatry in Stockholm. The symptom profile of the functional diagnosis (in accordance with the ROME-III criteria) was assessed in two steps: (1) self-administered online screening by the child, and (2) clinical interview with child and parent conducted by one of the study’s psychologists. During the clinical interview all inclusion and exclusion criteria were assessed and written informed consent was obtained from the parents. The children were asked if they wanted to participate in the study, but did not sign any consent form. Included participants were then scheduled for treatment sessions at the clinic.

All outcome measures were self-rated assessments that were administered online to both the children and their parents. Assessments were conducted at pre-treatment, post-treatment and 6-month follow-up. Parents were instructed to help their children if they needed assistance in completing the assessments, but not to influence the children’s answers. The children and parents had separate logins to the online platform. Clinical interviews were conducted to certify functional diagnosis after post and follow-up assessments. Participants were included in the study between May 2014 and January 2015. The last follow-up assessments were administered in November 2015.

This study was approved by the Regional Ethics Review Board in Stockholm, Sweden March 19, 2014 (2014/304-31/2) and registered at ClinicalTrials.gov in April 2014 (NCT02113605). The authors confirm that all ongoing and related trials for this intervention are registered in ClinicalTrials and have been approved by the Regional Ethics Review Board in Stockholm.

### Treatment

An overview of the content of the treatment is presented in Table 2. The treatment consisted of ten weekly sessions where the child and one or two parents worked together with the psychologist. In addition, there were seven sessions where the parents met with the psychologist without the child. Each session was 20–45 minutes long, depending on the content of the session and how much time the child and the parents needed. The two child psychologists who conducted all treatments (authors ML and MB) had previous experience of working with CBT for eight and ten years, respectively.

**Table 2:** Treatment content.

Session	Child and parent	Parent
1	Psychoeducation on abdominal pain. Goal setting.	Psychoeducation on modeling and reinforcement.
2	Thoughts and mindfulness. Mapping symptom-related behaviors.	Planning joyful activities with the child. Management of own feelings.
3	Building an exposure hierarchy. For children with IBS: toilet habits.	Supporting the child during exposure.
4	Psychoeducation on exposure. Functional analysis.	Praise and rewards. Functional analysis of parental behavior.
5	Follow up and plan new exposure exercises. Psychoeducation about fear and stress. Functional analysis.	Parental stress.
6	Follow up and plan new exposure exercises. Functional analysis.	Problem solving.
7	Follow up and plan new exposure exercises. Increasing difficulty in exposure. Functional analysis.	-
8	Follow up and plan new exposure exercises. Increasing difficulty in exposure. Functional analysis.	-
9	Follow up and plan new exposure exercises. Functional analysis. Quiz about the treatment.	-
10	Repetition, maintenance and relapse prevention.	Repetition, maintenance and relapse prevention.

The treatment was developed for this trial and builds on the exposure-based CBT-protocols for adults with IBS and adolescents with P-FGIDs designed by members of the research group [14, 16]. For the present study, examples and exercises were modified to fit younger children and parental involvement was emphasized. All children and parents received psycho-education about P-FGIDs and how avoidance and control behaviors are hypothesized to increase symptoms in the long run. Together with the psychologist, children assessed their behaviors related to avoidance and control of symptoms, conducted functional analyses of problematic behaviors, planned for exposure exercises that they engaged in together with their parents, and practiced mindfulness as a means to increase the effects of the exposure. Exposure exercises included going to school with an upset stomach and eating symptom-provoking food before engaging in activities. Parents learned about reinforcement principles and how to apply these, for instance by decreasing attention to their child’s pain behaviors. To promote positive interaction “golden moments” was introduced. During golden moments parents and children engaged in activities chosen by the child and were encouraged not to focus on the abdominal problems.

### Outcomes

#### Child-rated outcome measures

The primary outcome was child-rated pain intensity on the Faces Pain Rating Scale [23]. Secondary outcomes included: pain frequency measured by the question: “How many days during the last week did you have pain or discomfort?”; gastrointestinal symptoms measured by Pediatric Quality of Life Inventory Gastrointestinal Symptom Scale (PedsQL Gastro) [24]; quality of life measured by Pediatric Quality of Life Inventory (PedsQL QOL) [25]; somatic symptoms measured by Children’s Somatization Inventory (CSI-24) [26]; anxiety measured by Spence Children Anxiety Scale (SCAS) [27]; depressive symptoms measured by Child Depression Inventory (CDI) [28]; gastrointestinal symptom profile measured by a shortened version of ROME-III [2]; behavioral avoidance measured by IBS-behavioral responses questionnaire (IBS-BRQ) [29]; and satisfaction with the treatment measured by Client Satisfactory Questionnaire (CSQ) [30]. School absenteeism was examined with two questions. The first question (absence) assessed how many hours the child was absent from school due to pain, and the second question (leaving) assessed how many times in a month the child went home from school due to pain. These two questions replaced the full FDI questionnaire that originally was applied for in the ethical application [31].

The IBS-BRQ and CSQ measures are originally adult scales and wordings were changed to fit the pediatric population. One question about sexual activity was removed from IBS-BRQ.

#### Parent-rated outcome measures

The parented-rated assessments included parental versions of: PedsQL Gastro measuring the child’s gastrointestinal symptoms; PedsQL QOL measuring the child’s quality of life, and CSQ measuring the parent’s satisfaction with the treatment. Parental responses to children’s pain symptoms were measured by Adult Responses to Children’s Symptoms (ARCS) subscales Protect and Monitor, found to be sensitive to change [32, 33]. School absenteeism was assessed with parental versions of the children’s questions described above.

Because this was a pilot trial some additional measures were collected for exploratory purposes. These measures and the results for the measures are presented in Appendix 1.

### Data analysis

Two-tailed dependent *t*-tests were performed to detect significant within-group differences between pre- and post-treatment and between pre-treatment and 6-month follow-up. Maintenance of improvements and possible further improvements were investigated by comparing the post-treatment assessment with the 6-month follow-up assessment using dependent *t*-test. Effect sizes and 95% confidence intervals of changes between assessments were calculated as within-groups Cohen’s *d* [34] i.e., the standardized mean difference. Effect sizes were categorized according to Cohen's suggestion where small, medium, and large effect sizes are *d* = 0.20, 0.50, and 0.80, respectively [35]. In most cases, both parents completed the assessments (for 19 of 20 children) and the mean parent-rating was calculated for each child at each assessment point.

## Results

**Fig 1:**
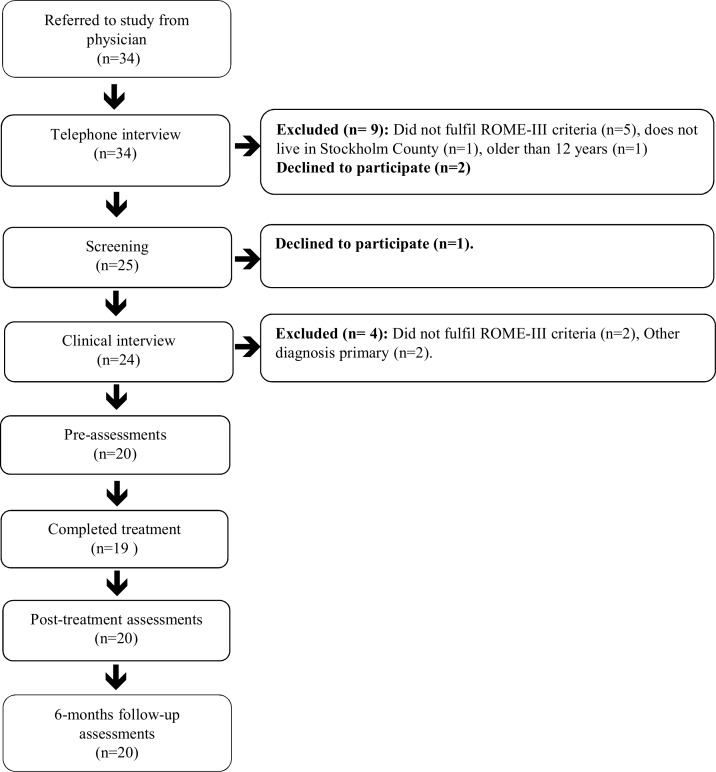
Participant flow through the study.

There were 34 children referred to the study. N = 11 were excluded and n = 3 declined to participate, see participants flow in Fig 1.

### Adherence and attrition

Children and their parents attended on average 9.3 (SD = 1.3) of 10 sessions. One child discontinued treatment after 5 sessions due to lack of motivation, but completed post- and follow-up assessments. The other 19 children attended between 8 and 10 sessions and were considered to be completers. All completers engaged in exposures to aversive stimuli and situations. The treatment had a once a week format, but due to holidays and cancellations most treatments lasted longer than 10 weeks. Mean length of treatment was 14.0 (SD = 3.4) weeks. After removing summer and Christmas holidays, the mean length of treatment was 12.0 (SD = 2.5) weeks (range 9–19 weeks). Mothers participated in 81% of the sessions and fathers in 51%. The overlap is due to sessions where both mothers and fathers attended. One ROME-questionnaire was missing at post-treatment and 6-month follow-up assessments and one report on school absenteeism was missing at post-treatment assessment.

### Outcome measures

As shown in Table 3, there were significant improvements on all measures but one from pre- to post-treatment. The results were maintained or further improved at 6-month follow-up.

**Table 3:** Results.

	Means and SDs			Effect sizes Cohen’s d (95% CI)		
Outcome measure	Pre	Post	FU6	Pre-post	Post-FU6	Pre-FU6
Child-reported variables:						
Faces	5.80	4.60	3.40	0.40*	0.41**	0.85**
	(2.97)	(3.05)	(2.68)	[-0.01, 0.81]	[0.00, 0.83]	[0.21, 1.48]
Pain freq.	4.85	3.20	2.35	0.68**	0.34	1.02***
	(2.32)	(2.50)	(2.56)	[0.32, 1.04]	[-0.02, 0.69]	[0.46, 1.58]
CDI	7.65	4.85	3.90	0.54*	0.23	0.69***
	(5.63)	(4.50)	(3.80)	[0.09, 0.99]	[-0.26, 0.72]	[0.35, 1.03]
SCAS	25.70	19.80	14.55	0.31*	0.35*	0.55***
	(20.09)	(15.81)	(11.81)	[0.02, 0.60]	[0.02, 0.69]	[0.23, 0.87]
School (absence)	1.45	0.68	0.65	0.71**	0.03	0.75**
	(1.10)	(1.06)	(1.04)	[0.28, 1.14]	[-0.25, 0.32]	[0.28, 1.22]
School (leaving)	0.65	0.47	0.45	0.22	0.03	0.26
	(0.81)	(0.77)	(0.69)	[-0.32, 0.77]	[-0.35, 0.42]	[-0.17, 0.70]
CSI-24	18.40	12.30	8.75	0.47**	0.40*]	0.66***
	(13.71)	(9.36)	(6.51)	[0.13, 0.81]	[0.07, 0.73	[0.33, 1.00]
IBS-BRQ	49.05	40.75	36.00	0.52*	0.44*	0.87**
	(18.40)	(11.75)	(9.00)	[-0.01,1.09]	[0.06, 0.81]	[0.19, 1.56]
PedsQL Gastr	61.81	71.91	80.69	0.69**	0.56**	1.44***
	(10.81)	(16.57)	(14.52)	[0.20, 1.18]	[0.19, 0.92]	[0.81, 2.06]
PedsQL QOL	80.37	84.84	89.46	0.36**	0.38*	0.83**
	(11.64)	(13.04)	(10.20)	[0.06, 0.65]	[0.01, 0.78]	[0.16, 1.49]
Parent-reported variables:						
School (absence)	1.90	0.75	0.95	1.01***	-0.19	0.83***
	(1.20)	(1.06)	(1.07)	[0.47, 1.56]	[-0.48, 0.10]	[0.35, 1.31]
School (leave)	0.55	0.30	0.43	0.42	-0.22	0.22
	(0.60)	(0.59)	(0.52)	[-0.07, 0.90]	[-0.63, 0.18]	[-0.08, 0.51]
ARCS protect	11.80	6.58	6.63	1.06***	-0.01	1.01***
	(5.22)	(4.59)	(4.97)	[0.58, 1.53]	[-0.35, 0.33]	[0.55, 1,48]
ARCS monitor	11.18	5.78	6.03	1.58***	-0.10	1.55***
	(2.95)	(2.58)	(2.30)	[0.82, 2.35]	[-0.54, 0.33]	[0.81, 2.30]
PedsQL Gastro	57.29	69.93	72.15	1.25***	0.18	1.34***
	(9.22)	(10.80)	(12.60)	[0.61, 1.90]	[-0.10, 0.47]	[0.55, 2.12]
Peds QL QOL	70.52	79.43	81.33	0.71***	0.14	0.84***
	(11.63)	(13.23)	(13.53)	[0.35, 1.06]	[-0.04, 0.33]	[0.50, 1.18]

Note: PedsQL Gastro and Peds QL QOL are reversely scored. Higher scores indicate improvement. Abbreviations: Faces = Faces Pain Rating Scale, Pain freq. = Pain frequency, PedsQL Gastro = Pediatric Quality of Life Inventory Gastrointestinal Symptom Scale, PedsQL QOL = Pediatric Quality of Life Inventory, CDI = Child Depression Inventory, SCAS = Spence Children Anxiety Scale, CSI-24 = Children’s Somatization Inventory, IBS-BRQ = IBS-behavioral responses questionnaire, ARCS = Adult Responses to Children’s Symptoms.

* = p< .05

** = p< .01

*** = p< .001

#### Primary outcome measure

We observed significant but small improvements at post-treatment on Faces, the child-rated primary outcome measure for pain intensity, *d* = 0.40. At 6-month follow-up the children reported statistically significant further improvement compared to post-treatment, *d* = 0.41, and the pre- to 6-month follow-up effect-size was large, *d* = 0.85.

#### Secondary outcome measures

Five of the secondary outcomes rated by the children showed a moderate effect size at post-treatment (pain frequency, gastrointestinal symptoms, depression, school absenteeism and avoidance behavior) and three had a small effect size (quality of life, anxiety and somatization). At 6-month follow-up, six of the nine child-rated outcomes showed statistically significant further improvement. Five of the pre-treatment to 6-month effect sizes were large (pain intensity, pain frequency, gastrointestinal symptoms, quality of life and avoidance behavior) and four were moderate (depression, anxiety, school absenteeism [absence] and somatization). Only three children reported no pain-related school absenteeism the month before treatment. At post and follow-up assessments, no pain-related school absenteeism during the last month was reported by 11 and 12 children, respectively. On the school absenteeism measure of leaving school during the day, we did not observe any significant effects at any assessment point.

Four of the parent-rated measures showed large effect sizes at post (child’s gastrointestinal symptoms, school absenteeism [absence], and parental protective and monitoring behaviors), and one showed a moderate effect size (child’s quality of life). Although we did not observe any significant further improvement from post-treatment to 6-month follow-up, all pre- to 6-month follow-up effect sizes on the parent-rated measures were large. No effects were observed on leaving the school during the day.

### Satisfaction and perceived usefulness

Mean score on the Client Satisfaction Questionnaire (max 27) was 21.9 (SD = 4.6) for the children and 23.9 (SD = 3.5) for the parents. All 20 children stated that the treatment had helped them deal more effectively with their symptoms and 19 of 20 were very (n = 13) or mostly (n = 6) satisfied with the treatment.

### Rome III criteria

At the screening assessment, two participants did not fulfill the Rome III criteria for P-FGIDs according to the self-administered questionnaire, but were found to fulfill the criteria for P-FGID at the subsequent clinical interview. At post-assessments five, (25%) of the 20 children did not meet P-FGID criteria according to the self-assessments on the ROME-III questionnaire and the clinical interview. At 6-month follow-up, twelve of the children (60%) did not fulfill the criteria on the questionnaire and at the clinical interview.

## Discussion

The main objective of this pilot study was to test feasibility, acceptability and potential efficacy of this newly developed exposure-based CBT protocol for children with P-FGIDs and their parents. Pain-inducing exposures may be very aversive, so the first question was: would the children engage in the exposure exercises? Nineteen of the 20 children did carry out exposures and completed between 8 and 10 sessions, indicating that an exposure-based approach is acceptable to children with P-FGIDs and their parents. This was strengthened by the fact that 19 of 20 children were satisfied with the treatment and all children perceived the treatment as helpful for dealing with their symptoms. Regarding potential efficacy, the results of this study showed significant reductions in pain, gastrointestinal symptoms, somatization, depression, anxiety and school absenteeism as well as an increase of the quality of life from pre to post-treatment. Results were maintained, and for most child-rated measures the effects were significantly larger at 6-month follow-up. Parental protectiveness and monitoring of the child’s symptoms significantly decreased and these results were maintained at 6-month follow-up.

The decreased school absenteeism shown in this study is an important result. School absenteeism is considered to be a serious public health issue and key risk factor for problems like psychiatric disorders and economic deprivation in adulthood [36]. Even though P-FGIDs are associated with increased school absenteeism, only a few previous CBT intervention studies have assessed this. Reductions in depressive and somatic symptoms as shown in this study may also be of specific importance since they have been linked to maintenance of abdominal problems into adulthood [5].

Most CBT protocols that have been proven effective for pain-related disorders in children use other strategies than exposures, such as coping strategies, relaxation and cognitive techniques [37]. Our study suggests that using exposures without adding any of these commonly used components may also lead to symptom reduction. Whether the treatment mechanisms in these different approaches are similar or not and which approach is most effective remains to be investigated.

Combining exposure-based CBT and parental interventions works well in treatments for children with anxiety disorders [38]. Considering the natural tendency to avoid aversive stimuli such as abdominal pain and the parental instinct to protect their children, we predicted that including parents in the treatment would be beneficial. The results of this study support this prediction.

The further improvements on the child-rated measures observed from post to follow-up as shown in this study could be explained by the sustained changes in parental responses to the children’s pain. It has been suggested that such changes in parental behavior may precede children’s symptom changes [33]. It is also possible that the delayed effects are in part caused by spontaneous recovery. However, for many children with P-FGIDs the problems are chronic [5] and in our study the children had suffered from P-FGID for a mean duration of 3.7 years before study entry. The results in this study are in line with the study by Bonnert et al on exposure-based CBT for adolescents with P-FGIDs, and other CBT-studies in the field [7, 16].

Strengths of the study include positive results on a wide range of measures. Triple informants were used in this study with data collection from children and, in most cases, two parents, and results were consistent between children and their parents. The participants were referred from primary, secondary and tertiary care units and diagnosed with P-FGID criteria by physicians and via a self-administered ROME-III formula verified in a clinical interview by the study psychologists. At post and follow-up diagnosis was assessed in the self-administered ROME-III formula and again verified in a clinical interview by the study psychologists. These recruitment and assessment procedures contribute to the external validity of the study. Measures were assessed online in the children’s homes without the influence of an assessor.

The most important limitation of the study is the within-group study design. With no comparison group, the study offered no control over other potential causes for the observed improvement, for example the passage of time, test-effects, and the effect of attention from a caregiver. The design precludes any firm conclusion about causality and the results should therefore be interpreted cautiously. Nevertheless, our study suggests that children with FGIDs and their parents are willing to participate in exposure-based CBT and show large adherence with the treatment. Furthermore, all results were based on self-administered instruments and it would be valuable to add some other source of information to increase the validity of the outcomes, for example attendance reports from the children’s schools. Parents were instructed to help the child with the assessments if necessary, but not to influence the answers. Despite this instruction we cannot preclude that parents may have, at least to some extent, influenced their child’s answers. The parents in the sample had a somewhat higher education than the source population; 64% had attended university, compared to 51% in the general adult population in Stockholm county [39]. This might have influenced the results since parents with a higher education may have more resources to help their children through the treatment. On the other hand, the parents’ educational level was not considered in the inclusion process which may indicate that the educational level seen in the sample is representative for families seeking this kind of intervention in a routine care setting.

In conclusion, the results in this study are promising and add to the previous positive results of exposure-based CBT for P-FGIDs in adolescents and adults. We believe that the specific focus on exposures and parental support contributes to the knowledge of which components are effective in CBT for P-FGIDs. The results need to be confirmed in a randomized controlled trial.

## Supporting Information

S1 Appendix Measures and results not included in the paper. (DOCX)

S2 Appendix Research plan to Ethics Committee in English. (DOCX)

S3 Appendix Change of earlier application to Ethics Committee in English. (DOC)

S4 Appendix Research plan to Ethics Committee in Swedish. (DOCX)

S5 Appendix Change of earlier application to Ethics Committee in Swedish. (DOC)
