# Supplementary material for: Exposure-Based Cognitive Behavior Therapy for Children with Abdominal Pain: A Pilot Trial
Source: PLoS One. 2016 Oct 13;11(10):e0164647. doi: 10.1371/journal.pone.0164647 (PMC5063361; doi:10.1371/journal.pone.0164647)
Supplement: S1 Appendix — (DOCX) [file pone.0164647.s001.docx]

**Appendix 1. Measures and results not included in the paper**

**Measures**

The assessments for children that are not included in the paper are: gastrointestinal symptoms measured by Gastrointestinal Symptom Rating Scale - IBS version (GSRS-IBS) (1) and pain response measured by Pain Response Scale (PRS) (2). GSRS-IBS is only validated for adults with IBS and PRS is not validated.

Parents were assessed on the following measures not presented in the paper: parental versions of CSI-24 (Children’s Somatization Inventory assessing somatic symptoms) (3) and SCAS (Spence Children Anxiety Scale ) (4). A scale on parental behaviors, PB-FGID (Parental Behavior Children’s Functional Gastrointestinal Disorders), developed by the research group, assessing frequency of parental behaviors that may influence the child to avoid symptoms and situations, was measured, as was the parents own gastrointestinal symptoms measured by the Gastrointestinal Symptom Rating Scale - IBS version (GSRS-IBS) (1).

**Table A.1. Results**

|  | Means and SDs | | | Effect sizes Cohen’s *d* (95% CI) | | |
| --- | --- | --- | --- | --- | --- | --- |
| Outcome measure | Pre | Post | FU6 | Pre-post | Post-FU6 | Pre-FU6 |
| Child-reported variables: |  |  |  |  |  |  |
| GSRS-IBS | 31.15  (11.55) | 25.30  (9.91) | 22.90  (8.01) | 0.54**  [0.13, 0.94] | 0.26  [-0.12, 0.64] | 0.79***  [0.31, 1.27] |
| PRS | 8.25  (6.83) | 5.65  (5.21) | 3.25  (3.89) | 0.42  [-0.14, 0.99] | 0.50**  [0.13, 0.86] | 0.89**  [0.17, 1.60] |
| Parent-reported variables: |  |  |  |  |  |  |
| CSI-24 | 13.83  (4.30) | 9.78  (4.60) | 10.23  (5.55) | 0.91***  [0.40, 1.41] | -0.09  [-0.47, 0.30] | 0.72*  [0.11, 1.33] |
| SCAS | 17.25  (7.41) | 15.18  (5.26) | 13.48  (5.96) | 0.29  [-0.01, 0.60] | 0.29*  [0.06, 0.53] | 0.54**  [0.22, 0.86] |
| PB-FGID | 13.28  (4.08) | 7.18  (3.96) | 6.43  (3.94) | 1.52***  [0.98, 2.05] | 0.19  [-0.08, 0.46] | 1.71***  [1.02, 2.39] |
| GSRS-IBS Parents | 25.80  (8.04) | 25.35  (8.10) | 25.93  (7.92) | 0.06  [-0.28, 0.40] | -0.02  [-0.50, 0.47] | -0.07  [-0.47, 0.33] |

Abbreviations: GSRS-IBS = Gastrointestinal Symptom Rating Scale IBS version, PRS = Pain Response Scale, CSI-24 = Children’s Somatization Inventory, SCAS = Spence Children Anxiety Scale, PB-FGID = Parental Behavior Functional Gastrointestinal Disorders.

* = p< .05, ** = p< .01, ***= p< .001

**References**

1. Wiklund IK, Fullerton S, Hawkey CJ, Jones RH, Longstreth GF, Mayer EA, et al. An irritable bowel syndrome-specific symptom questionnaire: development and validation. Scand J Gastroenterol. 2003 Sep;38(9):947–54.

2. Wicksell RK, Olsson GL, Hayes SC. Mediators of change in acceptance and commitment therapy for pediatric chronic pain. Pain [Internet]. 2011 Dec;152(12):2792–801. Available from: http://dx.doi.org/10.1016/j.pain.2011.09.003

3. Walker LS, Beck JE, Garber J, Lambert W. Children's Somatization Inventory: Psychometric Properties of the Revised Form (CSI-24). J Pediatr Psychol. 2009 Apr 22;34(4):430–40.

4. Spence SH, Barrett PM, Turner CM. Psychometric properties of the Spence Children's Anxiety Scale with young adolescents. J Anxiety Disord. 2003.
