# Supplementary material for: Exposure-Based Cognitive Behavior Therapy for Children with Abdominal Pain: A Pilot Trial
Source: PLoS One. 2016 Oct 13;11(10):e0164647. doi: 10.1371/journal.pone.0164647 (PMC5063361; doi:10.1371/journal.pone.0164647)
Supplement: S2 Appendix — (DOCX) [file pone.0164647.s002.docx]

Research plan in English (in Swedish for the Regional Ethics Committee)

# CBT for functional abdominal pain in children – a pilot study

## Background

Children with functional abdominal pain account for 10 % of al visits to pediatric clinics in Swedish [1] as well as international populations [2]. The pain-related functional gastrointestinal disorders are characterized by substantial, recurrent or persistent abdominal pain without an organic cause. The most common P-FGID diagnoses among children are functional abdominal pain (FAP), irritable bowel syndrome (IBS) and functional dyspepsia (FD) [3]. P-FGIDs are associated with an increased risk for anxiety and depression, high school absenteeism and health care consumption and low quality of life[ 2]. For many children the problems sustain into adulthood [4].

Research show limited support for pharmacological treatments as well as for dietary treatments [5,6]. On the other hand cognitive behavior therapy (CBT) has been shown to be promising for children with P-FGIDs [7,8]. The access to CBT for children with P-FGIDs is very limited because only a few CBT-therapists master treatments for these diagnoses [9].

One way of reaching more patients is through internet-delivered treatments. During the last years CBT has been proven effective for many disorders in adults, such as panic disorder, social phobia, depression, eating disorders tinnitus, headache chronic pain and insomnia [10]. At the unit for internet psychiatry in Stockholm more than 3000 patients have been treated witin public health care since 2007 (www.internetpsykiatri.se). For IBS in adults internet-based CBT with focus on exposures have been shown to decrease abdominal symptoms [11-13], be cost-effective [14] and give long-term effects [15]. Internet-based treatments with CBT have also been tried for children with good results on pain reduction [16,17]. However, in these studies the participants with P-FGIDs was very few, why it is difficult to draw conclusions for this group of patients.

In the present moment the research group are conducting a project to evaluate internet-based CBT for adolescents (ages 13-17) with P-FGIDs (EC approval nr 2011/1816-31/1) where we have treated 29 adolescents with P-FGIDs via the internet. Preliminary analyses shoe medium within-group effect sizes on symptoms and avoidant behavior (Cohen’s *d*>.50) and decreased worry about symptoms (Cohen’s *d*=0.73). These results are in line with those in studies of traditionally administered CBT for P-FGID [18]. At the present moment we are recruiting to a randomized controlled trial (EC approval nr 2013/911-31/1) where internet-delivered CBT for adolescents with IBS (n=100) are compared to a waitlist control. In parallel with this study the research group are now planning to develop and evaluate internet-based CBT for children 8-12 years with P-FGID.

### Aim

This study is a pilot study aiming to develop and test a CBT-based treatment protocol for children 8-12 years, later to be tested on the Internet. This is thus a pre-study before coming studies of internet-based CBT for younger children than the ones we treat today in our studies for adolescents. If this pilot study shows promising results we will submit a new application for testing the protocol, delivered over the internet.

### Question formulations

Is CBT an effective treatment method when it comes to decreasing symptoms and symptom-related worry and increase quality of life and function for children with pain-related functional gastrointestinal disorders?

### Significance

P-FGID is very common in children and adolescents and generates many health care contacts, though many health care providers don´t think they have much to offer. There is evidence that CBT is effective for P-FGID, but the availability is low or non-existing in Sweden. Internet-based CBT can increase the availability to health care for children and adolescents with P-FGIDs due to it´s advantages with increased geographical reach and less therapist time needed.

A pilot study like this is necessary to adjust the treatment content to the target group before being tested on the internet. Members of the research group have tried similar set-ups in pilot studies before within the development of treatment for IBS (EC approval nr 2005/1334 - 31/3) and hypochondria (EC approval nr 2007/737-31/1-4), which have resulted in effective and safe internet protocols for treatment. These pilot studies have been conducted in a way to be able to be published in scientific journals [11,19], which has contributed to the general development of knowledge. Further benefit of this study is that the participants may be helped by the treatment.

**Method**

*Participants*

The participants are recruited from child- and adolescents medical clinics within Stockholm County Council. All participants will be thoroughly assessed by their physician and keep this contact during the study. Assessment of inclusion criteria is carried out with a self-assessment formula and a clinical interview.

*Inclusion criteria*

Children between 8-12 years who are diagnosed by their physician of any of the diagnoses IBS, FD or FAP according to the ROME-III criteria can be included. Any medication has to be stable for at least three months. Parents and children have to speak Swedish and be willing to participate in a 2-3 months treatment with home assignments.

*Exclusion criteria*

Coexistence of a severe somatic diagnosis and/or if the child fulfills the criteria of other organic disorder that better explains the abdominal problems leads to exclusion. If the child fulfills criteria of a psychiatric diagnosis that is estimated to be more primary than the abdominal problems the child will be excluded and referred to a more suitable treatment.

Children with an ongoing psychological treatment will not be included. Participants can be excluded if child abuse, substance abuse in the family or custody fight is ongoing.

Children can be excluded during the study if exclusion criteria described above are discovered after the clinical interview.

### Data collection

Data from both children and parents are collected with self-assessment forms. Measures are conducted before and directly after the treatment. Follow-up data will be assessed 6 months after treatment. The self-assessment forms will be administered via the projects home page with a personalized login. These are the measures that will be used to measure treatment effects:

### Primary outcome measure

1. Pain intensity and frequency. Faces Pain Rating Scale. (FACES) [20].

### Secondary outcome measures

1. Gastrointestinal Symptom Rating Scale-IBS Version (GSRS-IBS) [21].
2. Gastrointestinal Symptom Scale (PedsQL Gastro) [22].
3. Somatic symptoms: Children´s Somatization Inventory (CSI 24) [23].
4. Function: Functional Disability Index (FDI) [24].
5. Pain reactivity: Pain Reactivity Scale (PRS) [25].
6. Behaviors to handle IBS: IBS-behavioral responses questionnaire (IBS-BRQ) [26].
7. Anxiety: Spence Children Anxiety Scale (SCAS) [27].
8. Depression: Child Depression Inventory (CDI) [28].

### Parents ratings:

Since the children are not very old and some may have problems administering the assessments the parents will also assess the measures above. The parents will also administer GSRS-IBS assessing their own abdominal symptoms. They will also assess two measures on problematic parental behaviors in response to children’s symptoms. Parental FGID (a new measure developed by the research group) and Adult responses to children’s symptoms (ARCS) [29].

*Sample size*

We plan on recruiting 20-30 children to the pilot study. This number is based on our previous experiences of pilot studies aiming to develop internet-based treatment protocols for adults [11,19]. This number of participants gives a picture of the expected heterogeneity in the patient group and how well treatment interventions can work for different patients. The sample size gives a 70-80 % power to detect significant treatment effects similar to the once that we observed in the internet treatment for adolescents, that is an effect size of Cohen’s *d*>.50, given an alpha level of .05. We consider this power acceptable in a study that mainly aims at developing the treatment protocol.

*Procedure*

The children and their parents are offered to participate in the study by the physician who assessed and diagnosed the child. Written information of the study (see appendix) will be distributed at the same occasion and the study contact person will then contact the families if they are interested in participating. At this first contact by the telephone a preliminary assessment is made of the family’s possibilities to be included. After this a psychologist conducts a clinical interview where obstacles for participating are investigated. On this occasion the family can ask any questions about the treatment and their participation in the study. Consent is obtained from both parents and the child on this occasion. If all parties agree that the treatment is suitable the family is included. Families who are excluded are told the reason for the decision and if needed are referred to other treatment.

The child gets 2-3 months treatment. The parents get parental training during the same period. Both parents and children will be asked for feedback on the exercises in the treatment, considering suitability and applicability. The experiences from the pilot study will be used to develop a written treatment protocol adjusted to children with P-FGID and their parents. The written material will be used in coming Internet studies.

*Treatment*

The treatment is being developed by the project group and is based in the well-tried internet-based CBT interventions of P-FGID for adults and adolescents. Licensed psychologists with long experience of CBT with children and adolescents develop the treatment content. Components in the treatment is exposure to decrease fear of symptoms and relaxation to gain control over stress that gives rise to more symptoms and parent training to give the parents tools to help their children in the treatment. The children and parents receive a detailed review of how excessive avoidance and controlling behaviors can maintain and even worsen the symptoms and decrease quality of life. Together with the psychologist the family analyze which types of avoidant and controlling behaviors using and how they worsen symptoms. Step-wise instructions are given on how the participants can expose to symptoms to decrease their learned fear of the symptoms. During the development of the treatment the treatment content will be evaluated and adjusted by the treating psychologists according to the experiences made.

*Earlier experiences of methods and access to relevant personnel*

According to a SBU-rapport (2005) there is strong evidence for the effects of CBT for children with syndromes related to anxiety. Some minor studies of CBT for P-FGID shows promising results. Our pilot study of internet-based CBT for adolescents shows promising results and for IBS internet-delivered CBT has been proven effective and cost effective. The neighboring research group, The child-internet project “Dare”, has conducted a pilot study (n = 30) and a randomized controlled study (n = 93) of internet-based CBT for anxiety disorders with promising effects.

This project is conducted in cooperation between The Institution of Clinical Neuroscience, Karolinska Institutet, the Child and Adolescent Psychiatry in Stockholm and the department of pediatric gastroenterology at Sachs’ Children’s Hospital. Brjánn Ljótsson from Karolinska Institutet who developed internet-delivered CBT for adults and co-started the Internet Psychiatry Unit in Stockholm is part of the research group. Ola Olén is child gastroenterologist and has written the national clinical guidelines for children with P-FGID. Eva Serlachius is a specialist in child and adolescent psychiatry and project leader for the Child Internet Project (BiP) where this study is conducted. BiP has approvals from the Etics Committee to conduct research on internet treatments for children and adolescents for the following studies: P-FGID (EC approval nr: 2011/1816 31/1), anxiety (EC approval nr: 2010/1993-31/4 2011 2027-31/5) and OCD (EC approval nr: 2012/1995-31/1). Erik Hedman Has conducted several randomized controlled trials on internet-baed CBT. He has also done research on prognostic and normative factors for psychological treatment and done health economical evaluations of internet-based CBT. The psychologists responsible for the treatment Maria Lalouni och Marianne Bonnert, have long experience of working with behavior change in children and adolescents in cooperation with parents, inter alia within the Komet-program for parents. Marianne Bonnert is the responsible psychologist in the parallel adolescent studies. All in all, the project group has expertise within internet-based treatment, treatments for behavior change and P-FGID in children and adolescents.

*Ethical considerations*

No negative effects are expected from the treatment or the study procedures. An uncontrolled study has a limited scientific value, which can be considered an ethical problem. However, since a treatment protocol for this population is missing we consider it to be necessary to conduct a pilot study to preconceive the effectiveness of the treatment. Considering the circumstances it is reasonable not to test a new protocol on more participants than necessary. If we gain positive results we will conduct a study where we try the protocol on the Internet.

A positive result could in the long run lead to a large group of patients gaining access to an effective treatment. Any ethical problem considering risk-benefit is hard to identify as the risk is estimated to be small and the potential benefit large.

The families are given written and verbal information of the study before written consent is obtained from the parents and verbal consent is obtained from the child. There is a risk that the children feel forced to participate when the parents give consent. This is counteracted by a clear declaration from the study psychologist that the child can decline to participate in the study and the treatment at any time, even if the parents still wants to continue. The families may discontinue their participation in the study at any time. This will not affect the families regular health care contact, as the study is conducted outside these.

**References**

1. Olén O, Uusijärvi A, Grimheden P. Vårdprogram, funktionella buksmärtor hos barn och ungdomar. Stockholms läns landsting; 2013 Feb pp. 1–50.

2. Chiou E, Nurko S. Functional abdominal pain and irritable bowel syndrome in children and adolescents. Therapy. 2011 May 1;8(3):315–31.

3. Rasquin A, Di Lorenzo C, Forbes D, Guiraldes E, Hyams JS, Staiano A, et al. Childhood functional gastrointestinal disorders: child/adolescent. Gastroenterology. 2006 Apr;130(5):1527–37.

4. Campo JV, Di Lorenzo C, Chiappetta L, Bridge J, Colborn DK, Gartner Jr JC, et al. Adult Outcomes of Pediatric Recurrent Abdominal Pain: Do They Just Grow Out of It? Pediatrics [Internet]. 2001;108(1):e1–e1. Available from: http://pediatrics.aappublications.org/cgi/doi/10.1542/peds.108.1.e1

5. Huertas-Ceballos AA, Logan S. Dietary interventions for recurrent abdominal pain (RAP) and irritable bowel syndrome (IBS) in childhood. Cochrane database of systematic reviews (Online) [Internet]. 2009;(1). Available from: http://onlinelibrary.wiley.com/doi/10.1002/14651858.CD003019.pub3/pdf/standard

6. Huertas-Ceballos AA, Logan S, Bennett C, Macarthur C. Pharmacological interventions for recurrent abdominal pain ( RAP ) and irritable bowel syndrome ( IBS ) in childhood ( Review ). Cochrane database of systematic reviews (Online). 2009;(1).

7. Huertas-Ceballos AA, Logan S, Bennett C, Macarthur C. Psychosocial interventions for recurrent abdominal pain ( RAP ) and irritable bowel syndrome ( IBS ) in childhood ( Review ). Cochrane database of systematic reviews (Online) [Internet]. 2009;(1). Available from: http://books.google.com/books?id=_on95iEZo1IC&pg=PA193&dq=Huertas+Ceballos&hl=&cd=17&source=gbs_api

8. Eccleston C, Tm P, Acdc W, Lewandowski A, Morley S. Psychological therapies for the management of chronic and recurrent pain in children and adolescents ( Review ). 2012 Sep 28;(2):1–52. Available from: http://onlinelibrary.wiley.com/doi/10.1002/14651858.CD003968.pub2/pdf/standard

9. Shafran R, Clark DM, Fairburn CG, Arntz A, Barlow DH, Ehlers A, et al. Mind the gap: Improving the dissemination of CBT. Beh Res Ther. 2009 Nov 1;47(11):902–9.

10. Hedman E, Ljótsson B, Lindefors N. Cognitive behavior therapy via the Internet: a systematic review of applications, clinical efficacy and cost–effectiveness. Expert Rev Pharmacoecon Outcomes Res. 2012 Dec;12(6):745–64.

11. Ljótsson B, Andréewitch S, Hedman E, Rück C, Andersson G, Lindefors N. Exposure and mindfulness based therapy for irritable bowel syndrome - an open pilot study. J Behav Ther Exp Psychiatry. 2010 Sep;41(3):185–90.

12. Ljótsson B, Falk L, Vesterlund AW, Hedman E, Lindfors P, Rück C, et al. Internet-delivered exposure and mindfulness based therapy for irritable bowel syndrome--a randomized controlled trial. Beh Res Ther. 2010 Jun;48(6):531–9.

13. Craske MG, Wolitzky-Taylor KB, Labus J, Wu S, Frese M, Mayer EA, et al. A cognitive-behavioral treatment for irritable bowel syndrome using interoceptive exposure to visceral sensations. Beh Res Ther. 2011 Jun 1;49(6-7):413–21.

14. Andersson E, Ljótsson B, Smit F, Paxling B, Hedman E, Lindefors N, et al. Cost-effectiveness of internet-based cognitive behavior therapy for irritable bowel syndrome: results from a randomized controlled trial. BMC Public Health. 2011;11:215.

15. Ljótsson B, Hedman E, Lindfors P, Hursti T, Lindefors N, Andersson G, et al. Long-term follow-up of internet-delivered exposure and mindfulness based treatment for irritable bowel syndrome. Beh Res Ther. 2011 Jan;49(1):58–61.

16. Palermo TM, Wilson AC, Peters M, Lewandowski A, Somhegyi H. Randomized controlled trial of an Internet-delivered family cognitive-behavioral therapy intervention for children and adolescents with chronic pain. Pain. 2009 Nov 1;146(1-2):205–13.

17. Hicks CL, Baeyer von CL, McGrath PJ. Online psychological treatment for pediatric recurrent pain: a randomized evaluation. J Pediatr Psychol. 2006 Aug;31(7):724–36.

18. Levy RL, Langer SL, Walker LS, Romano JM, Christie DL, Youssef N, et al. Cognitive-Behavioral Therapy for Children With Functional Abdominal Pain and Their Parents Decreases Pain and Other Symptoms. Am J Gastroenterol. Nature Publishing Group; 2010 Mar 9;:1–11.

19. Hedman E, Ljótsson B, Andersson E, Rück C, Andersson G, Lindefors N. Effectiveness and cost offset analysis of group CBT for hypochondriasis delivered in a psychiatric setting: an open trial. Cog Beh Ther. 2010 Dec;39(4):239–50.

20. Hicks CL, Baeyer von CL, Spafford PA, van Korlaar I. The Faces Pain Scale-Revised: toward a common metric in pediatric pain measurement. Pain [Internet]. 2001. Available from: http://ukpmc.ac.uk/abstract/MED/11427329

21. Wiklund I, Fullerton S, Hawkey C, Jones R, Longstreth G, Mayer EA, et al. An irritable bowel syndrome-specific symptom questionnaire: development and validation. Scand J Gastroenterol. 2003 Sep 1;38(9):947–54.

22. Varni JW, Lane MM, Burwinkle TM, Fontaine EN, Yossuef N, Schwimmer JB, et al. Health-Related Quality of Life in Pediatric Patients with Irritable Bowel Syndrome:: A Comparative Analysis. Journal of Developmental & Behavioral Pediatrics [Internet]. 2006 Dec 1;27(6):451. Available from: http://journals.lww.com/jrnldbp/Fulltext/2006/12000/Health_Related_Quality_of_Life_in_Pediatric.1.aspx

23. Walker LS, Beck JE, Garber J, Lambert W. Children's Somatization Inventory: Psychometric properties of the revised form (CSI-24). J Pediatr Psychol [Internet]. Soc Ped Psychology; 2009;34(4):430–40. Available from: http://jpepsy.oxfordjournals.org/content/34/4/430.short

24. Claar RL, Walker LS. Functional assessment of pediatric pain patients: psychometric properties of the functional disability inventory. Pain [Internet]. 2006 Mar;121(1-2):77–84. Available from: http://eutils.ncbi.nlm.nih.gov/entrez/eutils/elink.fcgi?dbfrom=pubmed&id= 16480823&retmode=ref&cmd=prlinks

25. Wicksell RK, Olsson GL, Hayes SC. Mediators of change in acceptance and commitment therapy for pediatric chronic pain. Pain. 2011 Dec;152(12):2792–801.

26. Reme SE, Darnley S, Kennedy T, Chalder T. The development of the irritable bowel syndrome-behavioral responses questionnaire. J Psychosom Res. 2010 Sep;69(3):319–25.

27. Spence SH, Barrett PM, Turner CM. Psychometric properties of the Spence Children's Anxiety Scale with young adolescents. J Anxiety Disord [Internet]. 2003;17(6):605–25. Available from: http://eutils.ncbi.nlm.nih.gov/entrez/eutils/elink.fcgi?dbfrom=pubmed&id= 14624814&retmode=ref&cmd=prlinks

28. Smucker MR, Craighead WE, Craighead LW, Green BJ. Normative and reliability data for the children's depression inventory. J Abnorm Child Psychol [Internet]. Kluwer Academic Publishers-Plenum Publishers; 14(1):25–39. Available from: http://link.springer.com/article/10.1007/BF00917219

29. Van Slyke DA, Walker LS. Mothers“ responses to children”s pain. Clinical Journal of Pain. 2006 May;22(4):387–91.
