# Supplementary material for: Exposure-Based Cognitive Behavior Therapy for Children with Abdominal Pain: A Pilot Trial
Source: PLoS One. 2016 Oct 13;11(10):e0164647. doi: 10.1371/journal.pone.0164647 (PMC5063361; doi:10.1371/journal.pone.0164647)
Supplement: S3 Appendix — (DOC) [file pone.0164647.s003.doc]

**Change of earlier application**

**EC approval nr:** 2014/304-31/2

**Title:** CBT for functional abdominal pain in children – a pilot study

**Representative:** Anders Ekbom, Karolinska Institutet; Charlotta Wiberg Spangenberg, Stockholms läns sjukvårdsområde.

**PI:** Ola Olén

**Payment by message ”Change FBA Olén”**

## About the approved study by the Ethics Committee

In the original application a measure for quality of life was missing. It was present in the appendix 5 in the parent’s measures but not in the children’s measures.

## Change of the application

The research group would like to ad the measure Pediatric Quality of Life Inventory (PedsQL)

*( Varni JW, Seid M, Kurtin PS. PedsQL 4.0: reliability and validity of the Pediatric Quality of Life Inventory version 4.0 generic core scales in healthy and patient populations. Med Care. 2001 Aug;39(8):800–12.).*

The measure is a self-assessment scale with 23 items. There is both a parental and a child version. The research group would like to ad this measure since it is urgent to assess how the parents and children experience if quality of life is changed by the treatment.

The research group believes that the proposed change does not affect the patient security in any way, while it contributes to the development of knowledge on how quality of life may be affected by the CBT treatment for P-FGID.

Supplemeted is Appenxix 5 *Measures:* CBT for functional abdominal pain in children – a pilot study with changes in yellow.

Signature from the researcher who conducts the project verifies that the research will be conducted according to the original application and this change of the application.

Place: Date:

_____________________________________________

Ola Olén, MD, Ass Chief Psysician

Dept of medicine, Unit of clinical epidemiology

Z5:00, Karolinska Universitetssjukhuset, Solna

17176 Stockholm

[ola.olen@ki.se](mailto:brjann.ljotsson@ki.se)
