# Supplementary material for: Exposure-Based Cognitive Behavior Therapy for Children with Abdominal Pain: A Pilot Trial
Source: PLoS One. 2016 Oct 13;11(10):e0164647. doi: 10.1371/journal.pone.0164647 (PMC5063361; doi:10.1371/journal.pone.0164647)
Supplement: S4 Appendix — (DOCX) [file pone.0164647.s004.docx]

Bilaga nr 2

Forskningsplan

# KBT för funktionell magsmärta hos barn – en förstudie.

## Bakgrund

Barn med funktionell magsmärta utgör 10 % av alla besök på pediatriska mottagningar i såväl svensk [1] som internationell population [2]. De smärtrelaterade funktionella magtarmsjukdomarna (P-FGID, pain predominant functional gastrointestinal disorders) karaktäriseras av kraftig, återkommande eller ihållande buksmärta utan somatisk skada eller sjukdom. De vanligaste P-FGID-diagnoserna bland barn är funktionell buksmärta (FAP), irritable bowel syndrome (IBS) och funktionell dyspepsi (FD) [3]. P-FGID är associerade med ökad risk för ångest och depression, hög skolfrånvaro och sjukvårdskonsumtion och en låg livskvalitet.[2]. För en stor andel kvarstår dessutom problemen upp i vuxen ålder [4].

Forskning visar på begränsat stöd för att farmakologisk behandling och olika typer av dieter leder till symtomlindring [5,6]. Däremot har kognitiv beteendeterapi (KBT) visat sig mycket lovande för barn med P-FGID [7,8]. Tillgången till KBT för funktionella magtarmproblem är dock oerhört begränsad då det finns få KBT-terapeuter som behärskar behandling för dessa diagnoser [9].

Ett sätt att nå ut till fler personer är internetbaserad behandling. Under de senaste åren har internetbaserad KBT visat sig vara effektivt för flera åkommor hos vuxna såsom paniksyndrom, social fobi, depression, ätstörningar, tinnitus, huvudvärk, kronisk smärta och insomni [10]. På Internetpsykiatrienheten, Psykiatri Sydväst (SLSO) har man sedan 2007 behandlat över 3000 patienter med depression, paniksyndrom, IBS och social fobi inom offentlig vård (www.internetpsykiatri.se). Vid IBS hos vuxna har internetbaserad KBT med fokus på exponering visat sig minska magtarmsymptom [11-13], vara kostnadseffektivt [14] och ge långsiktigt stabil förbättring [15]. Internetbaserad behandling med KBT har också prövats för barn med kronisk smärta med goda resultat på smärtreduktion [16,17]. I dessa studier var dock antalet deltagare med funktionell buksmärta mycket litet, varför det är svårt att dra slutsatser om effekten för just denna grupp.

För närvarande bedriver forskningsgruppen ett projekt för att utvärdera internetbaserad KBT för ungdomar (ålder 13-17) med P-FGID. Vi har i en pilotstudie (EPN dnr 2011/1816-31/1) behandlat 29 ungdomar med P-FGID via internet. Preliminära analyser visar medelstarka inomgruppseffekter på symtom och undvikandebeteenden (Cohen’s d>.50) och minskad oro för symtom (Cohen’s d=0.73). Dessa resultat är i linje med de som uppnåtts i studier av traditionellt förmedlad KBT vid P-FGID [18]. Just nu pågår rekrytering till en randomiserad studie (EPN dnr 2013/911-31/1) där internetbaserad KBT vid IBS hos ungdomar (n=100) jämförs med väntelista. Parallellt med denna tonårsstudie planerar forskningsgruppen nu att utveckla och utvärdera internetbaserad KBT för barn, ålder 8-12, med P-FGID.

### Syfte

Föreliggande ansökan avser en förstudie som syftar till att ta fram ett KBT-baserat behandlingsprotokoll för barn, ålder 8-12, som senare ska utprövas via internet. Detta är således en förstudie till kommande studier av internet-baserad KBT för yngre barn med P-FGID än de vi idag behandlar i våra tonårsstudier. Om förstudien visar lovande resultat inkommer vi med en ny etikansökan för att vidare utpröva behandlingen via internet.

### Frågeställningar

Är KBT vid smärtdominerad funktionell magtarmsjukdom hos barn en effektiv behandlingsmetod när det gäller att minska symtom och symtomrelaterad oro och öka livskvalitet och funktion?

### Betydelse

Funktionell buksmärta hos barn och ungdomar är mycket vanligt och ger upphov till många vårdkontakter, samtidigt som många vårdgivare inte tycker sig ha någon behandling att erbjuda. Det finns evidens för att KBT innebär effektiv hjälp för många med funktionella buksmärtor, men tillgängligheten är mycket låg eller icke existerande i hela landet. Internetbaserad KBT kan öka tillgängligheten till vård för barn och ungdomar med funktionella magtarmsjukdomar både geografiskt och tidsmässigt.

En förstudie av detta slag är nödvändig för att anpassa behandlingsinnehållet till målgruppen innan det utprövas via internet. Medlemmar i forskningsgruppen har tidigare prövat liknade upplägg med pilotstudier vid utveckling av behandling för IBS (DNR 2005/1334 - 31/3) och hypokondri (DNR 2007/737-31/1-4), vilket har resulterat effektiva och säkra internetbehandlingsprotokoll. Dessa pilotstudier har genomförts på sätt att de kunnat publiceras i vetenskapliga tidskrifter [11,19], vilket har bidragit till den generella kunskapsutvecklingen kring sjukdomarna. Ytterligare nytta med föreliggande studie är att de som deltar kan bli hjälpta av behandlingen.

**Metod**

*Studiedeltagare*

Studiedeltagare rekryteras från barn- och ungdomsmottagningar i Stockholms läns landsting. Samtliga deltagare kommer att vara utförligt utredda för sina symtom av läkare och behålla sin läkarkontakt. Bedömning om deltagare uppfyller inklusionskriterierna sker med hjälp av självskattningsformulär och klinisk intervju.

*Inklusionskriterier*

Ungdomar mellan 8-12 år som av läkare diagnosticerats med någon av diagnoserna IBS, FD eller FAP i enlighet med Rome III-kriterierna. Eventuell medicinering ska vara stabil sedan 3 månader. Föräldrar och barn behöver behärska svenska och vara villiga att delta i 2-3 månaders behandling med hemuppgifter.

*Exklusionskriterier*

Samtidig förekomst av allvarlig somatisk sjukdom och/eller om barnet uppfyller kriterierna för annan organisk sjukdom som bättre förklarar magtarmproblemen leder till exklusion från studien. Om barnet uppfyller kriterierna för psykiatrisk diagnos som bedöms som mer primär än magtarmproblemen exkluderas denna från studien och hänvisas till annan mer lämpad behandling.

Barn som har en pågående psykologisk behandling kommer inte att inkluderas. Vidare kan deltagare exkluderas vid pågående misshandel, övergrep eller missbruk i familjen samt vid pågående vårdnadstvist.

Exklusion kan även ske under pågående studie om uppgifter enligt ovan framkommer efter bedömningssamtalet.

### Datainsamling

Data från både barn och föräldrar kommer att samlas in i form av självskattningsformulär. Mätningar görs före och omedelbart efter avslutad behandling. Uppföljningsdata inhämtas 6 månader efter avslutad behandling. Självskattningsformulären kommer att fyllas i via projektets hemsida med hjälp av en personlig inloggningskod. Följande utfallsmått används för att mäta effekt av behandlingen:

### Primärt utfallsmått:

1. Smärtintensitet och frekvens: Faces Pain Rating Scale. (FACES) [20].

### Sekundära utfallsmått:

1. Gastrointestinal Symptom Rating Scale-IBS Version (GSRS-IBS) [21].
2. Gastrointestinal Symptom Scale (PedsQL Gastro) [22].
3. Somatiska symptom: Children´s Somatization Inventory (CSI 24) [23].
4. Funktionsnivå: Functional Disability Index (FDI) [24].
5. Reaktivitet på smärta: Pain Reactivity Scale (PRS) [25].
6. Beteenden för att hantera sin IBS: IBS-behavioral responses questionnaire (IBS-BRQ) [26].
7. Ångestnivå: Spence Children Anxiety Scale (SCAS) [27].
8. Grad av depression: Child Depression Inventory (CDI) [28].

### Föräldraskattningar:

Eftersom barnen är små och vissa kan ha problem med att fylla i självskattningsskalor kommer även föräldrarna att fylla även i ovanstående skalor om sina barn. De fyller även i symtomsskattningsskalan GSRS-IBS för sin egen del för att ge oss uppfattning om i vilken grad de själva besväras av magtarmsymtom. Utöver detta fyller de i två formulär som mäter problematiska föräldrabeteenden i respons till barnens symtom. Parental behavior FGID (ett nytt instrument utvecklat av forskargruppen under pilotstudien för ungdomar) och Adult responses to children’s symptoms (ARCS) [29].

*Sampelstorlek*

Vi planerar att rekrytera 20-30 barn till förstudien. Antalet bygger på våra tidigare erfarenheter av att genomföra förstudier med syfte att utveckla internetbehandlingsprotokoll för vuxna [11,19]. Detta antal ger en god bild av vilken heterogenitet man kan förvänta sig i patientgruppen samt hur väl behandlingsinterventioner fungerar för olika patienter. Powermässigt innebär sampelstorleken ca 70 % – 80 % power att ge signifikanta behandlingseffekter liknande de som vi observerat i studien av internetbehandling för ungdomar med P-FGID, dvs. effekt om Cohen’s d>.50, givet alphanivå om .05. Vi anser att denna power är acceptabel i en studie som främst avser att utveckla behandlingsprotokollet.

*Procedur*

Barnen och deras föräldrar erbjuds att delta i studien av barnets läkare som utrett och diagnosticerat barnet. Skriftlig försökspersonsinformation (se bilaga) utdelas vid samma tillfälle och familjerna kontaktas av projektets kontaktperson vid intresse av att delta. Vid denna första kontakt per telefon görs en preliminär bedömning av familjens möjlighet att inkluderas i studien. Bedömningssamtal med barn och föräldrar genomförs därefter med legitimerad psykolog där eventuella hinder för att delta i studien undersöks med en klinisk intervju. Familjen får också tillfälle att få information och ställa frågor om behandlingen och deras deltagande i studien. Skriftligt samtycke inhämtas från båda föräldrarna och barnet vid samma tillfälle. Om samtliga bedömer att behandlingen är lämplig inkluderas familjen. Familjer som exkluderas meddelas skälen till detta och vid behov hänvisning till annan behandling.

Barnet får under 2-3 månaders tid individuell behandling. Föräldrarna får under samma period föräldraträning. Såväl föräldrar som barn kommer kontinuerligt att tillfrågas om återkoppling på de övningar som ingår i behandlingen avseende begriplighet och tillämpbarhet. Erfarenheterna från förstudien kommer att användas för att ta fram ett skriftligt behandlingsmaterial anpassat efter barn med FGID och deras föräldrar. Det skriftliga materialet kommer sedan att användas i kommande internetstudier.

*Behandling*

Behandlingsinterventionen utvecklas av projektgruppen och är baserad på de beprövade internetbaserade KBT-behandlingarna av FGID för vuxna och tonåringar. Behandlingsinnehållet tas fram av legitimerade psykologer med lång erfarenhet av KBT för barn och ungdomar samt internetbehandling. Komponenter i behandlingen är exponering för att minska rädsla för symtom samt avslappning i syfte öka kontrollen över stressreaktioner som ger upphov till mer symtom samt föräldraträning för att ge föräldrarna verktyg att hjälpa sitt barn i behandlingen. Barnen och föräldrarna får en detaljerad genomgång av hur överdrivna undvikande- och kontrollbeteenden kan vidmakthålla och till och med förvärra FGID-symtom och minska livskvaliteten. Tillsammans med behandlare analyseras vilka typer av undvikande- och kontrollbeteenden just de ägnar sig åt och på vilket sätt de förvärrar symtomen. Instruktioner ges om hur de stegvis och på ett systematiskt sätt ska utsätta sig för symtom för att minska sin inlärda rädsla för symtomen. Under utvecklingen av behandlingen kommer behandlingens innehåll att löpande utvärderas och anpassas av de behandlande psykologerna utefter de erfarenheter som görs.

*Tidigare erfarenheter av metoder och tillgång till relevant personal*

Enligt en SBU-rapport (2005) finns det starkt forskningsstöd för effekten av kognitiv beteendeterapi hos barn och ungdomar med ångestrelaterade syndrom. Några mindre studier med KBT-behandling för funktionell magsmärta hos barn och ungdomar har visat på lovande resultat. Vår pilotstudie av internetbaserad KBT för ungdomar har visat på lovande resultat och vid IBS hos vuxna har internetbaserad KBT med fokus på exponering visat sig vara effektivt, både vad gäller behandlingseffekt och kostnadseffektivitet. Den angränsande forskargruppen, Barninternetprojektet Våga, har också genomfört en pilotstudie (n = 30) och en randomiserad kontrollerad studie (n = 93) av internet-KBT för barn (8-12) med ångestsyndrom med lovande effekter.

Projektet sker i samarbete mellan Institutionen för klinisk neurovetenskap, Karolinska Institutet, BUP-Divisionen (SLSO) och Gastrosektionen vid Sachsska Barnsjukhuset (SLL). I forskargruppen för denna studie ingår Brjánn Ljótsson från Karolinska Institutet, som utvecklade internet-KBT för vuxna med IBS. Han har också varit med och startat Internetpsykiatrienheten. Ola Olén är barngastroenterolog som bl.a. skrivit det nationella vårdprogrammet för patientgruppen. Eva Serlachius är specialist i barn-och ungdomspsykiatri och projektledare för BarnInternetProjektet (BiP) där denna studie genomförs. BiP har tidigare fått etiknämndens godkännande att bedriva forskning på Internetbehandling för barn och ungdomar genom följande studier: FGID (dnr: 2011/1816 31/1), ångest (dnr: 2010/1993-31/4 2011 2027-31/5) och tvångssyndrom (dnr: 2012/1995-31/1). Erik Hedman har genomfört flera randomiserade kontrollerade studier på Internetbaserad KBT. Han har också forskat om prognostiska och normativa faktorer för psykologisk behandling och gjort hälsoekonomiska utvärderingar av internetbaserad KBT. Behandlingsansvariga psykologer är Maria Lalouni och Marianne Bonnert, som har lång erfarenhet av att arbeta med beteendeförändring hos barn och ungdomar i samarbete med vårdnadshavarare, inom bl.a. KOMET-programmet. Marianne Bonnert är också ansvarig psykolog i de parallellt pågående tonårsstudierna. Sammantaget har projektgruppen expertis inom både internetbaserad behandling, behandling för beteendeförändring samt funktionell buksmärta hos barn och ungdomar.

*Etiska överväganden*

Inga negativa effekter bedöms föreligga av behandlingsprogrammet eller bedömningsförfarandet i sig. En okontrollerad studie innebär begränsningar av studiens vetenskapliga värde vilket kan betraktas som ett etiskt problem. Samtidigt saknas ett behandlingsprotokoll för denna population och vi anser att det är nödvändigt att först genomföra en förstudie för att bilda oss en uppfattning om behandlingens effektivitet. I så måtto är det etiskt rimligt att inte utsätta fler deltagare än nödvändigt för en potentiell mindre bra behandling. Om vi får positiva resultat kommer vi att genomföra en studie där vi prövar behandlingen via internet.

Ett positivt resultat från undersökningen skulle i förlängningen kunna innebära att en stor patientgrupp får tillgång till en effektiv behandling som kan erbjudas i storskalighet. Något etiskt problem vad gäller risk-nytta är i denna studie svår att identifiera då risken bedöms som liten och den potentiella nyttan som stor.

Familjerna ges både skriftlig och muntlig information om studien innan skriftligt samtycke inhämtas från både vårdnadshavarna och muntligt samtycke från barnet. Det finns en risk att barnen känner sig tvingade att delta om föräldrarna ger sitt samtycke. Detta motverkas genom att de informeras i live-kontakt och tydligt informeras om att deras negativa samtycke gäller även då föräldrarna vill delta. Familjen har när som helst rätt att avbryta sitt deltagande i studien. Då studien sker utanför reguljär vård kommer ett val att inte delta i studien inte att påverka de vanliga vårdkontakterna.

**Referenser**

1. Olén O, Uusijärvi A, Grimheden P. Vårdprogram, funktionella buksmärtor hos barn och ungdomar. Stockholms läns landsting; 2013 Feb pp. 1–50.

2. Chiou E, Nurko S. Functional abdominal pain and irritable bowel syndrome in children and adolescents. Therapy. 2011 May 1;8(3):315–31.

3. Rasquin A, Di Lorenzo C, Forbes D, Guiraldes E, Hyams JS, Staiano A, et al. Childhood functional gastrointestinal disorders: child/adolescent. Gastroenterology. 2006 Apr;130(5):1527–37.

4. Campo JV, Di Lorenzo C, Chiappetta L, Bridge J, Colborn DK, Gartner Jr JC, et al. Adult Outcomes of Pediatric Recurrent Abdominal Pain: Do They Just Grow Out of It? Pediatrics [Internet]. 2001;108(1):e1–e1. Available from: http://pediatrics.aappublications.org/cgi/doi/10.1542/peds.108.1.e1

5. Huertas-Ceballos AA, Logan S. Dietary interventions for recurrent abdominal pain (RAP) and irritable bowel syndrome (IBS) in childhood. Cochrane database of systematic reviews (Online) [Internet]. 2009;(1). Available from: http://onlinelibrary.wiley.com/doi/10.1002/14651858.CD003019.pub3/pdf/standard

6. Huertas-Ceballos AA, Logan S, Bennett C, Macarthur C. Pharmacological interventions for recurrent abdominal pain ( RAP ) and irritable bowel syndrome ( IBS ) in childhood ( Review ). Cochrane database of systematic reviews (Online). 2009;(1).

7. Huertas-Ceballos AA, Logan S, Bennett C, Macarthur C. Psychosocial interventions for recurrent abdominal pain ( RAP ) and irritable bowel syndrome ( IBS ) in childhood ( Review ). Cochrane database of systematic reviews (Online) [Internet]. 2009;(1). Available from: http://books.google.com/books?id=_on95iEZo1IC&pg=PA193&dq=Huertas+Ceballos&hl=&cd=17&source=gbs_api

8. Eccleston C, Tm P, Acdc W, Lewandowski A, Morley S. Psychological therapies for the management of chronic and recurrent pain in children and adolescents ( Review ). 2012 Sep 28;(2):1–52. Available from: http://onlinelibrary.wiley.com/doi/10.1002/14651858.CD003968.pub2/pdf/standard

9. Shafran R, Clark DM, Fairburn CG, Arntz A, Barlow DH, Ehlers A, et al. Mind the gap: Improving the dissemination of CBT. Beh Res Ther. 2009 Nov 1;47(11):902–9.

10. Hedman E, Ljótsson B, Lindefors N. Cognitive behavior therapy via the Internet: a systematic review of applications, clinical efficacy and cost–effectiveness. Expert Rev Pharmacoecon Outcomes Res. 2012 Dec;12(6):745–64.

11. Ljótsson B, Andréewitch S, Hedman E, Rück C, Andersson G, Lindefors N. Exposure and mindfulness based therapy for irritable bowel syndrome - an open pilot study. J Behav Ther Exp Psychiatry. 2010 Sep;41(3):185–90.

12. Ljótsson B, Falk L, Vesterlund AW, Hedman E, Lindfors P, Rück C, et al. Internet-delivered exposure and mindfulness based therapy for irritable bowel syndrome--a randomized controlled trial. Beh Res Ther. 2010 Jun;48(6):531–9.

13. Craske MG, Wolitzky-Taylor KB, Labus J, Wu S, Frese M, Mayer EA, et al. A cognitive-behavioral treatment for irritable bowel syndrome using interoceptive exposure to visceral sensations. Beh Res Ther. 2011 Jun 1;49(6-7):413–21.

14. Andersson E, Ljótsson B, Smit F, Paxling B, Hedman E, Lindefors N, et al. Cost-effectiveness of internet-based cognitive behavior therapy for irritable bowel syndrome: results from a randomized controlled trial. BMC Public Health. 2011;11:215.

15. Ljótsson B, Hedman E, Lindfors P, Hursti T, Lindefors N, Andersson G, et al. Long-term follow-up of internet-delivered exposure and mindfulness based treatment for irritable bowel syndrome. Beh Res Ther. 2011 Jan;49(1):58–61.

16. Palermo TM, Wilson AC, Peters M, Lewandowski A, Somhegyi H. Randomized controlled trial of an Internet-delivered family cognitive-behavioral therapy intervention for children and adolescents with chronic pain. Pain. 2009 Nov 1;146(1-2):205–13.

17. Hicks CL, Baeyer von CL, McGrath PJ. Online psychological treatment for pediatric recurrent pain: a randomized evaluation. J Pediatr Psychol. 2006 Aug;31(7):724–36.

18. Levy RL, Langer SL, Walker LS, Romano JM, Christie DL, Youssef N, et al. Cognitive-Behavioral Therapy for Children With Functional Abdominal Pain and Their Parents Decreases Pain and Other Symptoms. Am J Gastroenterol. Nature Publishing Group; 2010 Mar 9;:1–11.

19. Hedman E, Ljótsson B, Andersson E, Rück C, Andersson G, Lindefors N. Effectiveness and cost offset analysis of group CBT for hypochondriasis delivered in a psychiatric setting: an open trial. Cog Beh Ther. 2010 Dec;39(4):239–50.

20. Hicks CL, Baeyer von CL, Spafford PA, van Korlaar I. The Faces Pain Scale-Revised: toward a common metric in pediatric pain measurement. Pain [Internet]. 2001. Available from: http://ukpmc.ac.uk/abstract/MED/11427329

21. Wiklund I, Fullerton S, Hawkey C, Jones R, Longstreth G, Mayer EA, et al. An irritable bowel syndrome-specific symptom questionnaire: development and validation. Scand J Gastroenterol. 2003 Sep 1;38(9):947–54.

22. Varni JW, Lane MM, Burwinkle TM, Fontaine EN, Yossuef N, Schwimmer JB, et al. Health-Related Quality of Life in Pediatric Patients with Irritable Bowel Syndrome:: A Comparative Analysis. Journal of Developmental & Behavioral Pediatrics [Internet]. 2006 Dec 1;27(6):451. Available from: http://journals.lww.com/jrnldbp/Fulltext/2006/12000/Health_Related_Quality_of_Life_in_Pediatric.1.aspx

23. Walker LS, Beck JE, Garber J, Lambert W. Children's Somatization Inventory: Psychometric properties of the revised form (CSI-24). J Pediatr Psychol [Internet]. Soc Ped Psychology; 2009;34(4):430–40. Available from: http://jpepsy.oxfordjournals.org/content/34/4/430.short

24. Claar RL, Walker LS. Functional assessment of pediatric pain patients: psychometric properties of the functional disability inventory. Pain [Internet]. 2006 Mar;121(1-2):77–84. Available from: http://eutils.ncbi.nlm.nih.gov/entrez/eutils/elink.fcgi?dbfrom=pubmed&id= 16480823&retmode=ref&cmd=prlinks

25. Wicksell RK, Olsson GL, Hayes SC. Mediators of change in acceptance and commitment therapy for pediatric chronic pain. Pain. 2011 Dec;152(12):2792–801.

26. Reme SE, Darnley S, Kennedy T, Chalder T. The development of the irritable bowel syndrome-behavioral responses questionnaire. J Psychosom Res. 2010 Sep;69(3):319–25.

27. Spence SH, Barrett PM, Turner CM. Psychometric properties of the Spence Children's Anxiety Scale with young adolescents. J Anxiety Disord [Internet]. 2003;17(6):605–25. Available from: http://eutils.ncbi.nlm.nih.gov/entrez/eutils/elink.fcgi?dbfrom=pubmed&id= 14624814&retmode=ref&cmd=prlinks

28. Smucker MR, Craighead WE, Craighead LW, Green BJ. Normative and reliability data for the children's depression inventory. J Abnorm Child Psychol [Internet]. Kluwer Academic Publishers-Plenum Publishers; 14(1):25–39. Available from: http://link.springer.com/article/10.1007/BF00917219

29. Van Slyke DA, Walker LS. Mothers“ responses to children”s pain. Clinical Journal of Pain. 2006 May;22(4):387–91.
