# Supplementary material for: Exposure-Based Cognitive Behavior Therapy for Children with Abdominal Pain: A Pilot Trial
Source: PLoS One. 2016 Oct 13;11(10):e0164647. doi: 10.1371/journal.pone.0164647 (PMC5063361; doi:10.1371/journal.pone.0164647)
Supplement: S5 Appendix — (DOC) [file pone.0164647.s005.doc]

**Ändring av tidigare ansökan**

**Diarienummer:** 2014/304-31/2

**Titel:** Förstudie av KBT för barn med funktionell magtarmsjukdom

**Behörig företrädare:** Anders Ekbom, Karolinska Institutet; Charlotta Wiberg Spangenberg, Stockholms läns sjukvårdsområde.

**Forskare som genomför projektet:** Ola Olén

**Betalning sker med meddelande ”Ändring FBA Olén”**

## Om studien som godkänts av etikprövningsnämnden

I den ursprungliga ansökan fanns inte mätinstrument för att mäta barnens livskvalitet med. Dock fanns den med i Bilaga 5 under föräldrarnas mätningar, men inte under barnens mätningar.

## Önskade förändringar

Forskningsgruppen önskar lägga till måttet Pediatric Quality of Life Inventory (PedsQL)

*( Varni JW, Seid M, Kurtin PS. PedsQL 4.0: reliability and validity of the Pediatric Quality of Life Inventory version 4.0 generic core scales in healthy and patient populations. Med Care. 2001 Aug;39(8):800–12.).*

Mätinstrumentet är en självskattningsskala med 23 items som finns i både en barn- och en föräldraversion. Forskargruppen önskar lägga till skalan för barn och föräldrar då det är angeläget att undersöka hur de upplever att barnens livskvalitet påverkas av behandlingen.

Forskargruppens bedömning är att den föreslagna förändringen inte på något sätt påverkar patientsäkerheten för deltagarna medan den bidrar till kunskapsutvecklingen om hur barns livskvalitet kan påverkas av KBT-behandling för funktionell magtarmsjukdom.

Bifogad finns *Bilaga 5. Frågeformulär: Förstudie av KBT för barn med funktionell magtarmsjukdom* med ändringen gulmarkerad.

Undertecknad forskare som genomför projektet intygar härmed att forskningen kommer att genomföras i enlighet med ursprungsansökan och denna tilläggsansökan

Ort: Datum:

_____________________________________________

Ola Olén, Med Dr, Bitr Överläkare

Institutionen för medicin, Enheten för klinisk epidemiologi

Z5:00, Karolinska Universitetssjukhuset, Solna

17176 Stockholm

[ola.olen@ki.se](mailto:brjann.ljotsson@ki.se)
